# Carbon monoxide poisonings in hotels and motels: The problem silently continues

**DOI:** 10.1016/j.pmedr.2019.100975

**Published:** 2019-08-16

**Authors:** Neil B. Hampson, Kristina L. Hauschildt, Kayla Deru, Lindell K. Weaver

**Affiliations:** a Virginia Mason Medical Center, Seattle, WA, United States of America; b University of Washington School of Medicine, Seattle, WA, United States of America; c The Jenkins Foundation, Kelso, WA, United States of America; d Division of Hyperbaric Medicine Intermountain Medical Center, Murray, UT, United States of America; e Intermountain LDS Hospital, Salt Lake City, UT, United States of America; f University of Utah School of Medicine, Salt Lake City, UT, United States of America

**Keywords:** Carbon monoxide, Poisoning, Hotels, Carbon monoxide alarms

## Abstract

Carbon monoxide poisoning remains common in the United States. One component of effective prevention involves identification of scenarios in which poisoning occurs to guide development of appropriate interventions. This study was conducted to determine the significance of the problem of carbon monoxide poisoning occurring in US hotels, motels and resorts. This is a population-based case series of guests staying at US hotels, motels, and resorts from 2005 to 2018. Details of incidents and individuals poisoned with carbon monoxide were collected from online searches and professional experience of the authors. Data extracted included number of incidents and individuals poisoned, age of those poisoned, outcomes, source of carbon monoxide, and lodging type. From January 1, 2005 to December 31, 2018, 905 guests were poisoned in 115 identified incidents, including 22 fatalities. Children represented 16% of those poisoned and 27% of fatalities. Type of lodgings were hotels, motels, and resorts of all classes and located in a majority of states. Most poisonings were caused by natural gas fueled appliances and could likely have been prevented by an in-room carbon monoxide alarm. To reduce morbidity and mortality from unintentional CO poisoning in lodging facilities, government should mandate installation of in-room CO alarms, similar to the current requirement for smoke alarms.

## 1 Introduction

Unintentional carbon monoxide (CO) poisoning accounts for over 20,000 emergency department visits for non-fatal poisoning and approximately 400 deaths in the US annually (United States Centers for Disease Control and Prevention, 2008; Hampson, 2016). Poisoning is felt to be largely preventable through a triad of targeted education, warning labels on common CO poisoning sources, and use of CO alarms.

To pursue education for prevention, it is necessary to identify the sources and scenarios associated with poisoning and publicize them. More than a decade ago, Weaver and Deru reported the occurrence of CO poisoning at hotels, motels, and resorts (Weaver and Deru, 2007). They identified 68 incidents of accidental CO poisoning occurring from 1989 to 2004, involving 772 individuals, including 27 fatalities and 66 with confirmed neurological sequalae. They attempted to raise awareness of the problem and hoped to prevent future poisonings by encouraging regulations requiring CO alarms in these facilities.

Following a personal tragedy involving CO poisoning in a hotel, author KLH began collecting cases of CO poisoning in hotels and motels (ABC News, n.d.; Leland, 2014). Her lay effort prompted this report of ongoing poisoning incidents that are relatively unnoticed and unchecked.

## 2 Methods

Incidents involving CO poisoning that occurred in hotels, motels, and resorts were identified from internet searches, public media sources, and from professional experience of the authors. Because no single source identified all cases, a repeatable search strategy was not employed in the compilation of this clinical case series. Hard copies of full text articles were obtained for every incident identified online. Data extracted included location of incident, type of accommodation, source of CO, and number of individuals poisoned, stratified by fatal or nonfatal nature and age. Incidents since 2005 are reported here. Only victims evaluated by medical personnel or declared dead were classified as poisoned. Cases of intentional poisoning or poisoning from fire were excluded. Descriptive statistics were used for analysis.

## 3 Results

From January 1, 2005 to December 31, 2018, a total of 115 incidents of unintentional CO poisoning in US hotels, motels, or resorts were identified (Table 1). These occurred in 41 states. Number poisoned per incident ranged from 1 to 42 (median 21; mean ± 1 standard deviation 8 ± 8). A total of 905 individuals were poisoned, including 22 fatal poisonings (Table 1). Among survivors, 117 were known by the authors to have suffered permanent neurological injury. Children 18 years of age or younger accounted for 6 fatal and 143 nonfatal cases.

**Table 1:** Incidents of unintentional CO poisoning in hotels, motels and resorts by state, 2005–2018.

State	Incidents	# Poisoned		
		Total	Sequelae	Deaths
AL	1	15		
AR	2	15	4	
AZ	1	7		
CA	6	29	5	1
CO	3	23	4	
CT	1	2		
FL	7	33	7	6
GA	1	7		
IA	6	27		
ID	2	32		
IL	3	17	9	
IN	3	43		
KS	1	34		
KY	1	17	11	
LA	1	2		
MA	1	1		
MD	7	65	27	2
ME	3	29		
MI	3	18	2	1
MN	1	1	1	
MO	1	3		
MT	1	42		
NC	6	33	1	3
ND	3	31	3	
NH	1	12		
NJ	3	16		
NM	2	21	17	
NV	4	29		4
NY	3	12		
OH	2	39		
OK	1	21		
OR	1	3		
PA	7	61	2	1
TN	3	20		
TX	9	55	11	3
UT	3	11	4	
VA	3	16		
WA	1	2		
WI	4	35	2	
WV	3	26	7	1
	115	905	117	22

Seventy-six incidents (65%) occurred in facilities that were identified as members of national chains. Sources of CO are shown in Table 2. Most were fueled with natural gas.

**Table 2:** Incidents of unintentional CO poisoning in hotels, motels, and resorts by source of CO, 2005–2018.

	Usual Fuel	Incidents	Deaths
Boiler	Natural gas	24	1
Clothes dryer (hotel laundry room)	Natural gas	1	
Electrical generator	Gasoline	2	
Furnace	Natural gas	5	
"Heater"	Natural gas	3	
In-wall heater (guest room)	Natural gas	1	1
Motor vehicle exhaust (hotel garage)	Gasoline	1	5
Oven (hotel kitchen)	Natural gas	1	
Portable heater	Kerosene or propane	2	
Swimming pool heater	Natural gas	28	12
Water heater	Natural gas	22	3
"Multiple sources"	Natural gas	1	
Unspecified or unknown		24	
		115	22

## 4 Discussion

This case series details an ongoing risk for CO poisoning to guests staying at US hotels, motels and resorts. Such poisonings have continued over the past three decades, despite the interim development of a potential solution to the problem. It represents failure of both government and the lodging industry to protect the travelling public.

These poisonings occurred in a majority of states and in hotels of all classes, including those classified as “luxury.” It does not appear that one can avoid poisoning by paying more for lodging.

Most of the injuries and fatalities suffered by these individuals could likely have been prevented had CO alarms been installed in each hotel room, as smoke alarms have been since the Federal government mandated them in 1990 (Note, n.d.). Unfortunately, smoke alarms do not detect carbon monoxide and none of the incidents described in this series would have been expected to have been associated with smoke. CO has been called “the silent killer” because it is imperceptible to human senses.

Sources of CO in this series were located outside the guest room in all but one incident. The guest room was frequently located adjacent to or above the CO source, but this was not always the case. In some instances, the CO was determined to have originated floors away.

As of March 2018, 38 states required CO alarms in private dwellings, 27 via statute and 11 through adoption of the International Residential Code or via an amendment to their state's building code (National Conference of State Legislators, n.d.). However, only 14 states require them in hotels or motels under statute (California, Florida, Louisiana, Maine, Maryland, Michigan, New Jersey, New York, North Carolina, Oregon, Tennessee, Vermont, West Virginia, Wisconsin) (National Conference of State Legislators, n.d.). Where they are required, some states have attached a variety of qualifications and exclusions such as only “in newly constructed or repaired hotels,” only in rooms adjacent to a recognized source of CO, or “unless it is determined that no potential carbon monoxide hazard exists for that unit.” (National Conference of State Legislators, n.d.) The fact that CO can pass through drywall and potentially diffuse throughout a building raises the question of whether making the latter determination is possible (Hampson et al., 2013).

The lodging industry has opposed installation of CO alarms in guestrooms in the past on the basis of cost (Stoller, 2012). It is now possible to purchase a sealed residential CO alarm with a ten-year battery for under forty US dollars. Amortizing that price over ten years yields a cost of only about one cent per guestroom per day. Optimally, in the case of public lodgings, hardwired and centrally monitored combination smoke and CO alarms should be required for maximum safety.

The vacation rental industry has becoming an increasingly popular alternative to hotels and motels for short term vacation lodging and is expected to continue its growth (Orbis Research, n.d.). The rentals are listed on web-based sites and are convenient to book. The sites allow the user to search for rental features, including the presence or absence of a CO alarm in some cases. For example, each Airbnb lodging listing indicates whether the facility is equipped with a CO alarm and the brokerage company will provide an alarm for installation in each of their hosts' homes free of charge (Airbnb, n.d.).

Because CO poisoning is not a reportable condition and death certificate data reported by the National Center for Health Statistics only categorize place of death as medical facility, home, nursing home, hospice, or other (United States Centers for Disease Control and Prevention, National Center for Health Statistics, n.d.), tracking poisonings like these is quite difficult. The major limitation of this study is the potential for failure to identify incidents. A particular incident may not have been deemed newsworthy at the time it occurred, resulting in failure for it to be publicly reported. CO poisoning may not have been recognized by medical providers owing to its nonspecific symptoms. This potential for under counting is exemplified by the fact that 9 of the 115 incidents were identified only through the authors' own professional activity.

Under counting is also likely to have occurred in the case of cognitive sequelae. The media does not typically report such outcomes. A final limitation is the fact that this report does not include CO poisoning cases from the private vacation rental market.

As the traditional lodging industry (hotels, motels, and resorts) has not demonstrated inclination to install CO alarms in all guestrooms, government should mandate them as they do for smoke alarms. The technology has improved to the point where it is dependable, reliable, and inexpensive. There is an opportunity for a major hotel or motel chain to take up this initiative regarding public safety and lobby for alarm legislation while challenging other chains to do the same. Until rooms are outfitted with CO alarms, travelers should consider carrying one when staying in a facility not known to be so equipped (Hampson, 2009).

## Declaration of competing interest

The authors have no conflicts of interest to disclose.
